# MicroRNA Profiles Discriminate among Colon Cancer Metastasis

**DOI:** 10.1371/journal.pone.0096670

**Published:** 2014-06-12

**Authors:** Alessandra Drusco, Gerard J. Nuovo, Nicola Zanesi, Gianpiero Di Leva, Flavia Pichiorri, Stefano Volinia, Cecilia Fernandez, Anna Antenucci, Stefan Costinean, Arianna Bottoni, Immacolata A. Rosito, Chang-Gong Liu, Aaron Burch, Mario Acunzo, Yuri Pekarsky, Hansjuerg Alder, Antonio Ciardi, Carlo M. Croce

**Affiliations:** 1 MVIMG, Ohio State University, Columbus, Ohio, United States of America; 2 Dept. of Morphology, Surgery and Experimental Medicine, Universita' degli Studi, Ferrara, Italy; 3 UOSD of Clinical Pathology, Regina Elena Institute, Rome, Italy; 4 Dept. Experimental therapeutic-unit 1950, The University of Texas, MD Anderson Cancer Center, Houston, Texas, United States of America; 5 Dep. of Radiologic and Oncologic Sciences and Pathology, University of Rome “La Sapienza”, Rome, Italy; University of Barcelona, Spain

## Abstract

MicroRNAs are being exploited for diagnosis, prognosis and monitoring of cancer and other diseases. Their high tissue specificity and critical role in oncogenesis provide new biomarkers for the diagnosis and classification of cancer as well as predicting patients' outcomes. MicroRNAs signatures have been identified for many human tumors, including colorectal cancer (CRC). In most cases, metastatic disease is difficult to predict and to prevent with adequate therapies. The aim of our study was to identify a microRNA signature for metastatic CRC that could predict and differentiate metastatic target organ localization. Normal and cancer tissues of three different groups of CRC patients were analyzed. RNA microarray and TaqMan Array analysis were performed on 66 Italian patients with or without lymph nodes and/or liver recurrences. Data obtained with the two assays were analyzed separately and then intersected to identify a primary CRC metastatic signature. Five differentially expressed microRNAs (hsa-miR-21, -103, -93, -31 and -566) were validated by qRT-PCR on a second group of 16 American metastatic patients. *In situ* hybridization was performed on the 16 American patients as well as on three distinct commercial tissues microarray (TMA) containing normal adjacent colon, the primary adenocarcinoma, normal and metastatic lymph nodes and liver. Hsa-miRNA-21, -93, and -103 upregulation together with hsa-miR-566 downregulation defined the CRC metastatic signature, while *in situ* hybridization data identified a lymphonodal invasion profile. We provided the first microRNAs signature that could discriminate between colorectal recurrences to lymph nodes and liver and between colorectal liver metastasis and primary hepatic tumor.

## Introduction

The main cause of death in cancer patients is metastatic disease [1]. Cancer cells dissemination has been considered a late multi-step event during tumor development. After the primary tumor growth, genetically selected, malignant cells invade the local tissue, enter to blood and/or lymphatic vessels, are transported to distant sites and colonize new organ tissues. Recent evidences suggest that tumor spread could be an earlier event that will eventually manifest after several years from diagnosis [2]–[5].

Colorectal cancer is the third most common malignancy in the developed world after lung and breast cancer: about half of the patients die of metastatic disease within 5 years from diagnosis [6]. The first sites of metastatic disease of colorectal cancer are the regional lymph nodes and the liver. Pathological examination of colon adenocarcinoma can not accurately predict patients that will have disseminated disease to local lymph nodes and/or to distant sites. However, in colon, as well as in breast and melanoma cancer patients, the presence or the absence of lymph nodes invasion can influence the type of surgical resection or the type of chemotherapy regime [7]–[9].

Although metastases to the liver often come from colon cancers, there are cases where metastasis is the first and only finding in patients with an unknown primary tumor site [10], and the distinction between primary hepatic lesions and liver metastasis from different possible sites is of therapeutic and prognostic value [11].

Thus, there is an increasing need of novel diagnostic and prognostic biomarkers that could discriminate between the primary tumor and the different sites of metastasis, as well as predicting the metastasizing propensity of the primary tumor.

MicroRNAs or miRNAs, are small (19–25 nucleotides) non coding RNAs, that regulate genes expression by post-transcriptionally suppressing mRNA translation, and/or causing mRNA degradation. They are involved in the regulation of the majority of physiological processes [12], and are aberrantly expressed in tumor initiation and progression, predicting disease status and clinical outcome [13], [14]. Interestingly, microRNAs signatures are highly tissue specific and can be used to classify cancers, and to identify the primary tumor of a metastatic lesion of unknown origin [15]–[19].

The aim of our study was to identify a microRNA signature that could allow differentiating between lymphonodal and hepatic metastasis in patients with colo-rectal carcinoma.

## Materials and Methods

### Patient tissue samples

Three groups of patients were considered in the study (Table1).

Paraffin embedded tissues coming from 66 patients diagnosed with colonic carcinoma were selected from the the University of Rome “La Sapienza”, UOC Anatomia ed Istologia Patologica C/Cardiovascolare, tissue bank according to the TNM staging.

Out of 66 patients, 18 presented a primary colonic tumor with no metastasis (Any T, N0, M0), 33 had colon cancer with lymph nodes metastasis only (Any T, Any N, M0) and 15 were diagnosed with colon cancer, lymph nodes and liver metastases (Any T, Any N, M1). Separate tumor samples from the primary tumor, the metastatic lymph nodes and the liver metastasis were collected for each patient and processed for tissue microarray and TaqMan Array MicroRNA Cards.

A second group of 16 patients coming from the files of the OSU Pathology Department were enrolled in the study. For each patient, normal colonic tissue, primary colonic adenocarcnoma, metastatic lymph nodes and metastatic liver were analyzed, as well as the corresponding normal lymph nodes and liver, when available. The TMA that was generated from these patients samples plus three sets of US Biomax Tissue arrays (BN05014 with 24 cases, BC05118 with 50 cases, CO702 with 69 cases) were used for In Situ Hybridization(ISH). US Biomax cases represent the third group of patients included in the study with normal adjacent colonic tissues, primary colon adenocarcinoma with no lymph node/distant metastases and primary colon cancer of patients with documented lymph nodes and liver metastasis, and the actual lymph node and/or liver metastases.

The project was approved by the Italian (Presidente - Prof. Aldo ISIDORI, Prof. Lucio MIANO, Dott.ssa Amalia ALLOCCA, Dott.ssa Enrica ARDUINI, Dott.ssa Maria CAPORALE, Prof. Luciano CAPRINO, Dott.ssa Avia CARABELLI, Prof. Francesco COGNETTI, Dott.ssa Anna DALLE ORE, Prof.ssa Marzia DUSE, Dott.ssa Giuseppina DI GIAMMARCO, Dott. Domenico Antonio IENTILE, Prof. Giovanni FABBRINI, Prof.ssa Paola FRATI, Dott.ssa Maria Teresa LUPO, Prof. Franco MANDELLI, Dott. Enrico MARINELLI, Dott.ssa Boza MAURO, Prof. Paolo MENE', Dott.ssa Elisabetta SIMONGINI, Prof. Pietro SERRA, Prof. Giovanni SPERA, Dott. Ettore TIBERI, Avv. Angelo TUZZA, Prof.ssa Annarita VESTRI, Prof. Vincenzo ZIPARO) and American (http://orrp.osu.edu/irb/irbforms/) Ethical Commettee (Prot.1119/13, Prot.2007E0748 respectively). Specifically, both the Commettees, the Ohio State University Institutional Research Board and il Comitato Etico “La Sapienza”, exempted us from patients informed consent since, in both cases, paraffin embedded tissue were anonymously archived samples, selected according to their TNM staging and patients were not longer available.

### RNA extraction

Total RNA was extracted processing five 20 µm thick sections from each paraffin embedded tissue, using the RecoverAll Total Nucleic Acid Isolation Kit (AMBION #1975) and following manufacturer's instructions.

RNA labeling and hybridization on miRNA microarray chips were performed as previously described [20]. Five µg of each sample total RNA was hybridized on our miRNA microarray (Ohio State Comprehensive Cancer Center, version 2.0), which contains 460 mature miRNAs probes, spotted in quadruplicate (235 *Homo sapiens*, 222 *Mus musculus*, and three *Arabidopsis thaliana*). Different probes were used to recognize each mature miRNA and most of the miRNAs precursors. Hybridization signals were detected with Streptavidin Alexa Fluor 647 conjugates and scanned images (Axon 4000B) were quantified using the Genepix 6.0 software (Axon Instruments). The same samples were also analyzed with RT-PCR cards (Applied Biosystems).

### miRNA microarray

Microarrays were essentially analyzed as described by Liu et al [21]. Quantiles normalization and statistical analysis was performed using BRB ArrayTools developed by Richard Simon and Amy Peng Lam [22].

Differentially expressed miRNAs were identified using a random-variance t-test. The random-variance t-test is an improvement over the standard separate t-test as it permits sharing information among genes about within-class variation without assuming that all genes have the same variance. Genes were considered statistically significant if their P-value was lower than 0.05. [23].

Multiple testing was controlled using false detection rate. miRNA nomenclature was established according to the UCSC Genome Browser (http://genome.ucsc.edu/) and the miRBase (http://www.mirbase.org/); in case of discrepancies the miRBase was followed.

Raw data are available on the GEO website (http://www.ncbi.nlm.nih.gov/geo/query/acc.cgi?acc=GSE56350).

### TaqMan Array MicroRNA Cards and Quantitative Real Time PCR

Real Time PCR was performed using the TaqMan Array Human MicroRNA panel (v.1, Applied Biosystems, Foster City, CA) and 50 ng of RNA per port for a total of 400 ng. This array contains 365 miRNA targets as well as endogenous controls. Normalization was performed with small nuclear RNAs (snRNA) U44 and U48. Expression of single mature miRNAs was assessed by the TaqMan miRNAassay (Applied Biosystems, Foster City, CA, USA), and normalized to RNUB6 (Applied Biosystems). Raw data are available on the GEO website (http://www.ncbi.nlm.nih.gov/geo/query/acc.cgi?acc=GSE56350).

### In situ hybridization

In situ hybridization was performed for selected microRNAs following a previously described protocol [24], using Exiqon LNA-5'DIG labeled probes. Three USBiomax tissue microarrays (TMA) slides (Colon normal Tissue array with 21 normal cases and 3 adenocarcinoma cases, Colon cancer and matched adjacent normal tissue array with 25 cases, Colon cancer tissue array with metastasis and adjacent normal tissue with 68 cases) and a slide with tissue cores from 16 patients (OSU TMA) were used. For each selected microRNA, TMAs were scored according to the relative microRNA expression of each core by two pathologists as defined by the percentage of positive tumor cells and the strength of the signal in these cancer cells in positive cases. The scores were generated blinded to the presence or absence of metastatic disease and the Mann-Whitney test was applied to compare classes.

## Results

Our study was developed in four steps by taking advantage of: (I) DNA Microarrays, (II) Taqman high density cards, (III) Taqman single assay qRT-PCR and (IV) in situ hybridization. To achieve more robust results, we used three different groups of patients (Table 1). The first group included 66 Italian patients (III Clinica Chirurgica, Universita' di Roma “La Sapienza”, Rome, Italy) with colonic adenocarcinoma: 18 patients did not have any metastasis, 33 had affected lymph nodes and 15 showed metastases at both lymph nodes and liver. Total RNAs from 18 colonic primary tumors (T) with no metastasis, 33 colonic primary tumors (T_N_) with lymphonodal recurrences and the corresponding 33 metastatic lymph nodes (N_N_), 15 colonic primary tumors (T_L_,) of patients with lymphonodal and hepatic metastatic disease and the corresponding 15 metastatic lymph nodes (N_L_) and liver lesions (L), were tested on the custom microarray platform that had been previously used to establish cancer microRNA signatures in several studies [13], [14], [21], and on Taqman Array microRNA Cards (Applied Biosystems). Data were normalized according to the platform requirements and the same comparisons among classes were applied to both the datasets separately. Significantly altered miRNAs were identified and results were intersected. Only microRNAs with a fold change difference larger than 2 or lower than 0.5 were considered as the putative pro-metastatic and anti-metastatic miRNAs respectively. Twelve microRNAs were consistently up-regulated in metastasis (hsa-miR-103, -155, -16, -191, -200c, -21, -24, -26a, -26b, -29a, -31 and 93) and only two were down-regulated (hsa-miR-328 and -566) (Table 2).

**Table 1:** Patients grouping and TNM classification.

Assays	Cases	Patients	TNM		
**RNA Microarray (I)**	66	18	AnyT	N0	M0
**&**		33	AnyT	AnyN	M0
**TaqMan Array (II)**		15	AnyT	AnyN	M1
**RT-PCR (III)**	16	16	AnyT	AnyN	M1
**IV TMA**					
**OSU TMA**	16	16	Normal Colon		
		6	Normal Lymph Nodes		
		4	Normal Liver		
		16	Adenocarcinoma		
		10	Positive Lymph Nodes		
		12	Liver Metastasis		
**USBIOMAX TMA**	24	21	Normal Colon		
**BN05014**		3	Adenocarcinoma		
**USBIOMAX TMA**	24	21	Normal Colon		
**BC05118**					
**USBIOMAX TMA**	68	3	Normal Colon		
**C0702**	+1 marker	3	Normal Lymph Nodes		
		3	Normal Liver		
		30	Adenocarcinoma		
		24	Positive Lymph Nodes		
		5	Liver Metastasis		

**Table 2:** Experimental procedure and miRs signatures.

Assay	Upregulated Has-MiRs	Downregulated Has-MiRs
**RNA Microarray &**	103, 155, 16, 19, 200c, 21,	328, 566
**TaqMan Array**	24, 26a, 26b, 29a, 31, 93	
**RT-PCR**	103, 21, 31, 93	566
**TMAs**	103, 21, 93	566

The double array strategy allowed us to test the same samples with two different technical approaches.

To further confirm the identified “metastatic”signature, selected microRNAs were tested on sixteen American patients with metastatic colon cancer (OSU Pathology Dept., Columbus, OH). For each patient, total RNA of paraffin embedded tissue cores of normal and malignant colon, lymph node and liver, was assayed by single qRT-PCR. The Mann-Whitney test was applied to compare the PCR results from the different patient groups.

**Figure 1:**
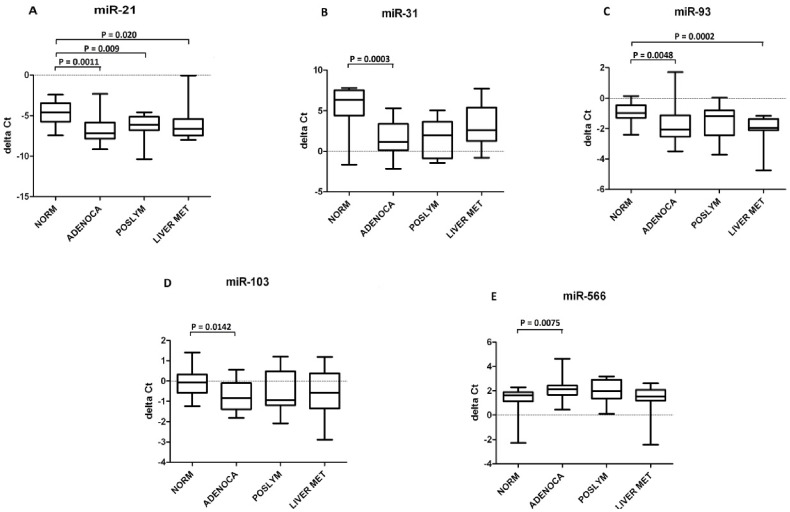
qRT-PCR Box-Plots. Normal colon tissue (NORM), colon adenocarcinomas (ADENOCA), lymphonodal metastasis (POSLYM) and colon liver (LIVERMET) for miR-21(A), miR-31 (B), miR-93 (C), miR-103 (D) and miR-566 (E). A Mann-Whitney test was applied to compare groups. Groups are shown on the boxplots' x-axis, while the delta Ct Values are represented on the y-axis. For each box, the bar represent the Median, the area the 25^th^ and 75^th^ percentile and the whiskers of the graph the largest and smallest values. Each P-value bar correspond to a comparison: for each miR the first lower bar refers to the NORM vs ADENOCA comparison; miR-21 second lower bar P-value corresponds to the NORM vs POS LYMPH comparison, while the first upper bar of both miR-21 and -93 corresponds to the NORM vs LIVER MET comparison.

Primary metastatic colonic adenocarcinomas showed a significant overexpression of hsa-miR-21 (P-value = 0.0011), -31 (P-value  = 0.0003), -93 (P-value  = 0.0048), -103 (P-value  = 0.0142) and downregulation of hsa-miR-566 (P-value  = 0.0075) when compared to their adjacent normal tissue (Table 2). Moreover, respect to normal, hsa-miR-21 and -93 were significantly upregulated in liver metastasis with a P-value = 0.020 and P-value = 0.0002 respectively. Hsa-miR-21 was also significantly overexpressed in metastatic lymph nodes (P-value = 0.009) (Fig.1). These five validated microRNAs did not correlate with tumor staging.

**Figure 2:**
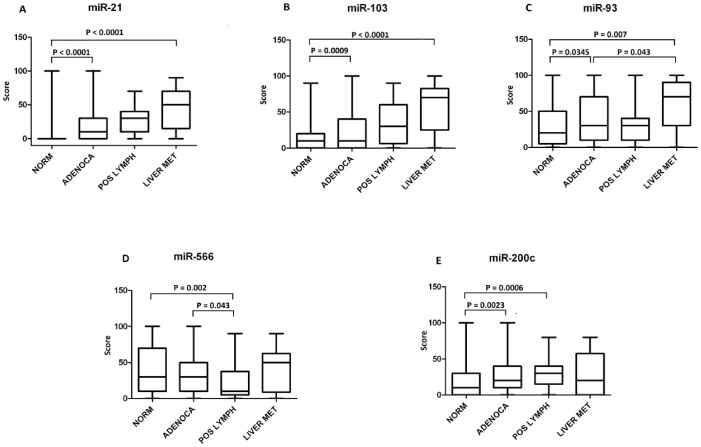
TMA Box-Plots. with normal colon tissue (NORM), colon adenocarcinomas (ADENOCA), lymphonodal metastasis (POSLYM) and colon liver (LIVERMET) for miR-21 (A), miR-103(B), miR-93(C), miR-566(D) and miR-200c(E). A Mann-Whitney test was applied to compare groups. Groups are shown on the boxplots' x-axis, while the average score Values are represented on the y-axis. For each box, the bar represent the Median, the area the 25^th^ and 75^th^ percentile and the whiskers of the graph the largest and smallest values. Each P-value bar correspond to a comparison: for miR-21,-103,-93, and -200c the first lower bar corresponds to the NORM vs ADENOCA comparison; miR-21,-103, and -93 first upper bars refer to the NORM vs LIVER MET comparison, while miR-566 and -200c upper bar shows the NORM vs POS LYMPH comparison; miR-566 and miR-200c lower bars indicate the ADENOCA vs POS LYMPH and the NORM vs POS LYMPH comparison respectively.

**Figure 3:**
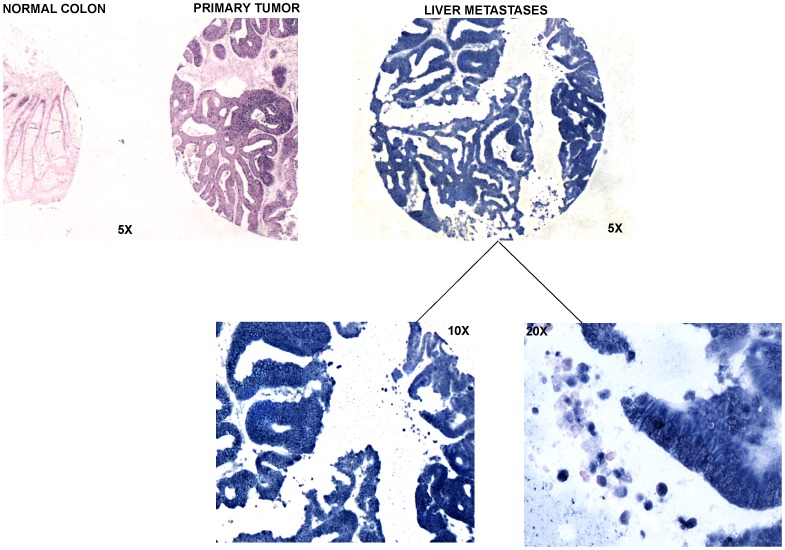
miR-21 In Situ Hybridization of Normal Colon (5X), Primary Tumor (5X) and Liver Metastasis (Different magnifications: 5X, 10X, 20X). As documented in literature, miR-21 showed a weak signal in normal colon respect to adenocarcinoma (P-value = 0.0011) and it was highly expressed in metastasis. MiR-21 expression was detected only in primary adenocarcinoma and metastatic malignant cells.

**Figure 4:**
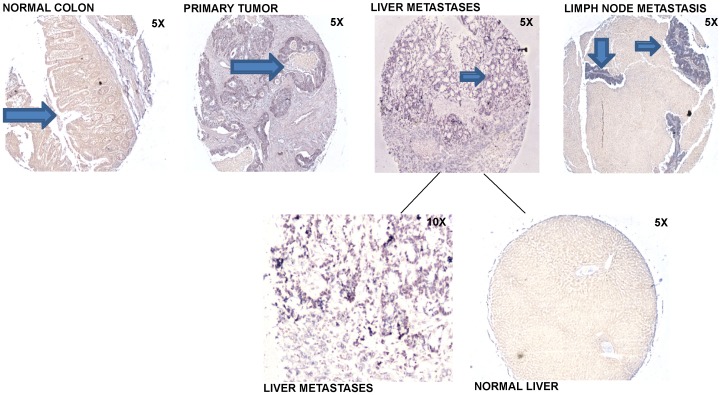
miR-103 In Situ Hybridization of Normal Colon (5X), Primary Tumor (5X), Normal Liver (5X), Liver Metastasis (5X, 10X) and Metastatic Lymph node (5X). In normal colon the arrow is pointing at the lack of miR-103 expression in epithelial cells. In the primary tumor the arrow is pointing at the increased expression of miR-103 in the invasive adenocarcinoma. The arrow in liver recurrence and the two arrows in the lymph node metastases are showing a dramatic increased expression of miR-103 in the metastatic adenocarcinoma epithelia.

**Figure 5:**
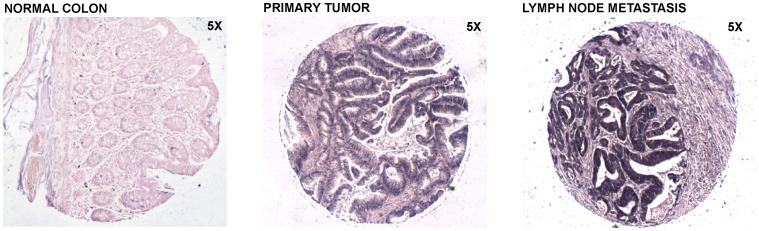
miR-93 In Situ Hybridization of Normal Colon (5X), Primary Tumor (5X) and lymph node metastasis (5X).

In tumors, the inflammatory and stromal cells are scattered among benign and malignant epithelial cells, and variably contribute to the cellularity of the lesion. It is, thus, essential to understand which type of cell expresses a specific microRNA. For this purpose, in situ hybridization experiments were carried out on TMAs with primary adenocarcinomas, metastatic lymph nodes, hepatic metastasis and normal tissues. Hsa-miR-21, -93, -103 and -566 probes were hybridized onto OSU TMA and USBiomax TMA slides. Two independent pathologists scored each slide core blinded to the site of the core biopsy and to the clinical staging history, considering the signal intensity of colonic malignant cells compared to the adjacent normal epithelial and non-epithelial cells (Fig.2). Hsa-miR-21 (P-value <0.0001) and -103 (P-value  = 0.0009) were significantly over-expressed in adenocarcinomas and metastatic cancers when compared to normals (Fig.2: A, B). A Chi-squared test revealed a linear trend for both, hsa-miR-21 (P-value<0.0001) and hsa-miR-103 (P-value<0.0001): they were increasingly upregulated progressing from normal, to adenocarcinoma and to metastasis (Fig.3, Fig 4). However, while hsa-miR-103 was significantly overexpressed in liver metastasis when compared to adenocarcinomas and positive lymph nodes, hsa-miR-21 did not show any significant difference among the three classes. ISH of hsa-miR-93 confirmed its upregulation in adenocarcinomas and liver metastasis, showing a significant difference between normals and either adenocarcinomas (P-value  = 0.0345) or liver metastasis (P-value  = 0.007) and between adenocarcinomas and liver metastasis (P-value  = 0.043). No significant difference was found when comparing normals and adenocarcinomas versus positive lymph nodes (Fig. 2C and Fig.5). Thus, hsa-miR-93 expression pattern could differentiate normal colon from primary adenocarcinoma, and normal colon and primary adenocarcinoma from liver metastasis, but not from secondary tumoral lymphnodal invasion. Hsa-miR-566 was equally overexpressed in normal adjacent colonic tissue, primary adenocarcinoma malignant cells and liver colonic metastatic cells (Fig. 2D). However, positive lymph nodes showed a significantly weaker signal in respect to the normals (P-value  = 0.002) and adenocarcinoma (P-value  = 0.043), suggesting that loss of hsa-miR-566 may facilitate lymph node invasion in colon cancer.

Induction of Epithelial-to-Mesenchimal Transition (EMT) has been considered an important step in the development of metastatic cancer. Recently, it has been demonstrated that hsa-miR-103/107 indirectly downregulates miR-200 family members, and that such pattern is associated with mesenchymal phenotype in breast cancer cell lines [24].

In our study, the microarray platform and Taqman assay, isolated both hsa-miR-103 and hsa-miR-200c. We wanted to understand whether, as in breast metastatic cancer, these two miRNAs were inversely correlated in colon metastatic cancer. Although not significant by qRT-PCR, TMAs were hybridized with hsa-miR-200c probe and scored (Fig. 2E). Compared to normals, a higher signal intensity was detected in adenocarcinomas (P-value  = 0.0023) and positive lymph nodes (P-value  = 0.0006). A significant positive correlation between hsa-miR-103 and hsa-miR-200c was present in lymph node metastasis (P-value  = 0.0225), but not in adenocarcinomas or liver metastasis. Therefore, hsa-miR-103 and hsa-200c were not inversely correlated and do not regulate EMT in metastatic colon cancer *in vivo*, but were both overexpressed in adenocarcinomas and lymph nodes metastasis. We did not investigate hsa-miR-107 or any other members of the miR-200 family because EMT was not relevant to the aim of our study.

Interestingly, hsa-miR-31 ISH (data not shown) revealed a very strong signal in inflammatory cells surrounding the tumors. A weaker staining was observed in tumor and normal colonic cells, but only few hsa-miR-31 expressing lymphocytes were present in normal lymph nodes, suggesting that hsa-miR-31 upregulation resulted mostly from the inflammatory rather than from the malignant component of the tumor.

Table 1 reports the number of cases with TNM staging that were analyzed at each technical step of our study; Table 2 shows the microRNA signatures obtained with each technical procedure.

## Discussion

The 2009 AJCC edition has published a new colon cancer TNM classification, in which, subclasses have been added to the (N) nodal disease staging. Other authors have proposed to replace these N categories with the number of positive lymph nodes, and several surgical publications have suggested a more radical lymphadenectomy to better define prognosis in order to improve colon cancer patients survival [26]–[29]. Nodal invasion is a critical step for defining colon cancer patients prognosis and therapy. However, 25% of lymph node-negative patients experience recurrence and not all patients with positive lymph nodes have a poor prognosis [30]–[32].

On the other hand, primary tumor spread to the liver, is the major cause of disease progression in colorectal cancer patients, with 15–30% of patients presenting with synchronous hepatic lesions at the time of primary colorectal surgery, and, showing metachronous recurrences after aggressive liver resection [33]–[35].

Thus, it is essential to identify new molecular biomarkers that, together with the TNM staging system, can improve the diagnosis and classification of metastatic colon cancer patients.

The aim of this study was to identify a microRNA signature for metastatic colon cancer that could discriminate between lymph nodes and liver metastasis.

In literature, most of the reported microRNAs cancer signatures have been identified and validated on the same tissue samples. To increase the reliability of our data, with the exception of the first two analyses, each validation step was performed on a different group of patients.

Microarray and cards platforms intersected data identified fourteen differentially expressed microRNAs, of which five were validated by qRT-PCR. Hsa-miR-21, -31, -93, -103 were upregulated in primary tumors compared to normal, whereas hsa-miR-566 was downregulated. Hsa-miR-93 and -31 were also upregulated in liver metastasis, but not in lymph nodes positive for metastasis, whereas hsa-miR-21 over-expression was evident in both, liver and lymph nodes metastasis. With the exception of hsa-miR-31, in situ hybridization (ISH) of colonic adenocarcinomas and the corresponding adjacent normal tissue, as well as its nodal and liver metastasis tissue microarrays, confirmed the signatures identified by the cards and qRTPCR analyses. Hsa-miR-31 has been found dysregulated in several primary epithelial tumors where, according to tumor localization, it seems to trigger different cancer pathways: it is up-regulated in some carcinomas [36]–[41], and down-regulated in others [42]–[45]. Such a duality, has been found in invasive tumors as well: in colon cancer cell lines, invasion is promoted by hsa-miR-31 overexpression [46], [47], while, in breast cancer cell lines, its low levels promote a more aggressive behavior [42], which correlates to a poorer patient prognosis.

Targeted functions of hsa-miR-31 include angiogenesis, where its upregulation increases blood, but not lymphatic vessels sprouting [48], and acute inflammation [49]. We found that hsa-miR-31 is mostly up-regulated in peritumoral inflammatory cells, compared to cancer and normal cells. Thus, hsa-miR-31 dysregulation in primary aggressive tumors is an indisputable evidence, but the interplay of its functions between the inflammatory, neoangiogenic and tumoral metastatic context, needs, still, to be elucidated.

Hsa-miR-21 is another multitask microRNA that has been implicated in inflammation [50]–[53], immune disease [54]–[57], development [58]–[61], angiogenesis [62], [63], cancer and metastasis [18], [64]–[82]. Several studies have, in fact reported its over-expression in different type of tumors, including colorectal carcinoma, where its levels correlate to clinical progression, poor survival and therapeutic outcome [83], [84].

In agreement with our previous studies and those of others, qRT-PCR validated hsa-miR-21 upregulation in colonic adenocarcinomas and metastasis when compared to normal. In situ hybridization data showed a trend: hsa-miR-21 levels were the lowest in normal tissue, and increasingly higher in adenocarcinoma and metastasis, meanwhile, a similar expression was observed in cancer cells nodes and liver recurrences, suggesting that hsa-miR-21 upregulation could predict an advanced metastatic disease.

As with hsa-miR-31, high levels of hsa-miR-21 and -93 have been described by other authors in hepatocellular carcinoma [39], [85], [86] and, therefore, they can not be considered as a diagnostic differential signature between liver metastasis and hepatocellular carcinoma.

The ISH data for hsa-miR-93 completely overlapped the qRT-PCR results: an intense staining was detected in primary colonic adenocarcinomas and liver metastasis. Induced over-expression of hsa-miR-93 was shown: to be tumorigenic in vitro and in vivo, to promote survival but not proliferation, and to enhance neoangiogenesis [87]. Moreover, increased hsa-miR-93 levels, correlate to progression and decreased patients survival in serous ovarian carcinoma [88] and, can predict relapse breast cancer [89].

However, the only up-regulated validated microRNA identified in our signature that could discriminate between colonic hepatic metastasis and primary hepatocellular carcinoma was hsa-miR-103. The relevance of this miRNA in cancer has been assessed in pancreatic, bladder, esophageal, gastric and breast tumors [29], [90]–[93]. High levels of hsa-miR-103 were correlated to poor survival in esophageal cancer and are associated to metastatic disease in breast and gastric cancer. Particularly, hsa-miR-103 upregulation was detected in gastric cancer patients bearing positive lymph nodes. Similarly, in our study, qRT-PCR detected hsa-miR-103 over-expression in primary tumors as well as metastatic nodes and liver. Again, ISH gave us more specific cell-of-origin information: while, there were no significant differences between primary tumors and positive lymph nodes, liver metastasis showed the highest levels of hsa-miR-103 expression.

In our study, although not validated by qRT-PCR, hsa-miR-200c was one of the selected microRNA by microarray and cards platforms. Recently, hsa-miR-103/107 levels were found inversely correlated to hsa-miR-200c in the regulation of Epithelial-to-Mesenchimal Transition in breast cancer [25]. To test if we could establish the same correlation in colon cancer, we performed ISH of colon TMA with miR-200c probe. We did not observe any inverse correlation between the two miRNAs. They were both over-expressed, but, hsa-miR-200c was strongly up-regulated in lymph nodal metastasis compared to normal colon, adenocarcinomas and liver recurrences. This suggests that hsa-miR-103 and hsa-miR-200c do not control EMT in colon cancer, and that high levels of hsa-miR-200c are required for nodal invasion.

Hsa-miR-200c liver metastasis ISH scores showed high variability, covering the average values of normal tissue, adenocarcinomas, and positive lymph nodes. Downregulation of hsa-miR-200c has been proposed as a distinctive signature of primary liver cancer (hepatocellular carcinoma, intrahepatic cholangiocarcinoma and hepatic angiosarcoma) from liver metastasis of epithelial origin [86], [94], while its upregulation in colon adenocarcinoma has been associated to a poor prognosis [95]. In our study, hsa-miR-200c upregulation in nodal metastasis was confirmed by ISH, but was not validated by qRT-PCR. This finding is due mainly to experimental and technical procedures. First, we used different groups of patients for validation, which, although providing more reliable end data, introduced more variability into the experimental procedure. Second, RNAs coming from our first microarrays patient group, were not extracted from microdissected paraffin embedded tissues and contained variable proportions of cancer and benign surrounding tissue, while the RNAs and tissues analyzed in the qRT-PCR and ISH were tissue microdissected cores with a major cancer component. However, qRT-PCR cannot differentiate from benign and/or non cancer cells, whereas ISH can discriminate among the different types of cells. Other authors validated their data on the same patients, and RNA was extracted from the whole tissue. Moreover, to the best of our knowledge, in literature there is no study reporting such a large scale of microRNA ISH. We have demonstrated that ISH can provide very useful cell specific information, suggesting additional microRNAs *in vivo* functions for further investigations.

An important novelty of our signature was hsa-miR-566. Apparently down-regulated only in adenocarcinomas with respect to controls and metastatic colonic tissues by qRT-PCR, hsa-miR-566 digoxigenin labeled probe, was weakly detected in positive lymph nodes cancer cells, and equally expressed in normal colon, as well as adenocarcinomas and liver metastasis, supporting the notion that ISH can give us more informative details than microarrays or qRT-PCR. Unfortunately, in literature, there is no study reporting hsa-miR-566 involvement in cancer, or describing its biological functions, that could aid the discussion of our data. We can only state that hsa-miR-566 was down-regulated in nodal colon metastatic cancer.

Several microRNA signatures based on clinical outcomes, have been identified to predict prognosis, but, so far, none of them has reported a distinctive pattern between nodal and liver recurrences.

We identified a colon metastatic signature that can discriminate between lymph nodes and liver metastasis. High levels of hsa-miR-21, -93 and -103 characterized liver metastasis, and thus, a more advanced metastatic disease, whereas, an over-expression of hsa-miR-200c coupled with low levels of hsa-miR-566, could be able to detect nodal invasion. Future stratified studies need to be conducted in order to ratify and correlate our signature to prognostic clinicopathologic factors.
